# The Importance of Common Currency Tasks in Translational Psychiatry

**DOI:** 10.1007/s40473-021-00225-w

**Published:** 2021-02-12

**Authors:** Alexandra C. Pike, Millie Lowther, Oliver J. Robinson

**Affiliations:** 1 Anxiety Lab, Neuroscience and Mental Health Group, University College London Institute of Cognitive Neuroscience, Alexandra House, 17-19 Queen Square, Bloomsbury, London, WC1N 3AR UK; 2 Research Department of Clinical, Educational and Health Psychology Department, University College London, Gower Street, London, WC1E 6BT UK

**Keywords:** Common currency tasks, Translational tasks, Translational psychiatry, Validity, Animal models, Behavioural assay

## Abstract

**Purpose of Review:**

Common currency tasks are tasks that investigate the same phenomenon in different species. In this review, we discuss how to ensure the translational validity of common currency tasks, summarise their benefits, present recent research in this area and offer future directions and recommendations.

**Recent Findings:**

We discuss the strengths and limitations of three specific examples where common currency tasks have added to our understanding of psychiatric constructs—affective bias, reversal learning and goal-based decision making.

**Summary:**

Overall, common currency tasks offer the potential to improve drug discovery in psychiatry. We recommend that researchers prioritise construct validity above face validity when designing common currency tasks and suggest that the evidence for construct validity is summarised in papers presenting research in this area.

## Introduction

The rate of drug discovery in psychiatry has not met expectations for a number of years [1, 2], with particular failings in translating promising pre-clinical findings into humans. This state of affairs can be attributed, in part at least, to discrepant findings from human and animal research [3]. Specifically, the relevance of pre-clinical work to human disease or symptoms is constrained by the similarities between measures used in humans and other species [3]. One way to improve this situation may be to use more ‘common currency’ tasks, which investigate the same phenomenon in different species. In this review, we will define common currency tasks and discuss their benefits for translational psychiatric research. Then, we will summarise some recent research using common currency tasks, and finally present some promising future avenues and our recommendations for this area.

## Defining Common Currency Tasks

A common currency task is one which has been designed to measure the same construct across species: key aspects are maintained when the task is performed by both humans and animals, although some features of the task may be altered to account for differences between species (for example, the range of auditory frequencies that can be perceived differs substantially between humans and rodents). There may also be more marked differences between species: a human completing the CANTAB spatial working memory task must explore and remember on-screen visuospatial information, whilst a rat completing a radial arm maze is required to physically move through space [4]. Some of these differences are driven by the direction of translation of the specific task. Common currency tasks may have originated in humans and been simplified for their translation into animal work (reverse or back-translation, e.g. the intra-extra dimensional set shift task from the CANTAB battery [5, 6]). Alternatively, they may have originated in animals, and the context may have been changed to allow translation into humans (forward translation [7]). Some tasks have been translated multiple times between species (e.g. the ‘ambiguous-cue interpretation’ task [8–10]), leading to multiple species-specific alterations.

Regardless of apparent similarities or differences between tasks, it is a non-trivial problem to ensure that all versions of a task are measuring the intended construct. Existing recommendations for the development of common currency tasks include minimal verbal instructions, non-verbal stimuli, similarity of task parameters (such as number of trials and stimulus timing) and required responses, as well as consistent statistical analyses following data collection [11], all of which influence the translational validity of common currency tasks.

## Translational Validity

**Fig. 1:**
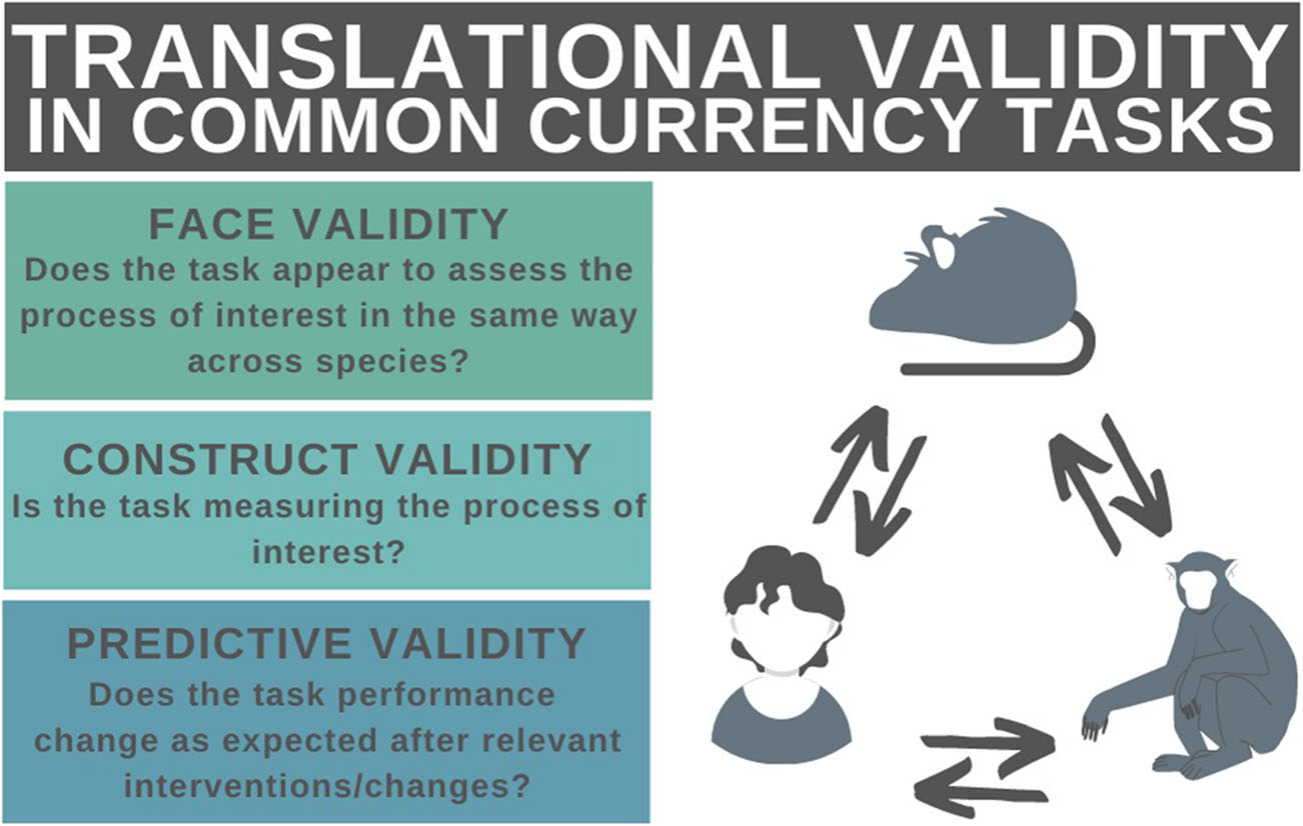
Graphic displaying the components of translational validity, which may be defined as the extent to which tasks designed to capture the same phenomenon in different species achieve this goal, along with their descriptions

The extent to which tasks are truly ‘common’ is known as ‘translational validity’ [12, 13]. Translational validity is composed of multiple types of validity, including face validity, predictive validity and construct validity (Fig. 1). Face validity is the degree of phenomenological similarity between test contents and a construct (e.g. whether the task appears, at face value, to be assessing working memory [14]). Predictive validity is the ability of a measure derived from a task to predict a subsequent score or outcome, such as response to treatment (e.g. whether the number of items recalled in a working-memory task increases after administration of a pro-cognitive drug [14]). Construct validity is the extent to which a task actually probes the intended underlying variable (e.g. working memory [15]).

### Face Validity

Face validity is arguably the easiest component of translational validity to assess. If two tasks appear similar, then it increases confidence that they are measuring the same thing and that success on the task is not driven by different strategies between species. However, high face validity does not guarantee construct validity—for example, humans and rodents may use spatial strategies to different extents when completing the Morris water maze, despite the use of virtual reality in humans to promote face validity [16]. Face validity may therefore be a red herring—researchers may focus too closely on making sure the task appears similar in both species, and less time focusing on whether it requires the same strategies and processes and is implemented using similar neural circuitry [12].

In particular, it is common for tasks in humans and animals to use different reinforcers—primary reinforcers such as food or water are often used in animal work, and secondary reinforcers such as money or points are often used in human work. Whilst this reduces face validity, it can be argued to increase construct validity. On the one hand, using money or points across species would likely evoke little reward-seeking behaviour in animals, as they do not have the same learnt associations between money and primary reinforcers as humans do. On the other hand, using food is unlikely to elicit strong responses from humans who generally have sufficient access to food, and food-restricting humans to ensure that they are sensitive to primary reinforcers (which is routine in animal experiments) would be difficult due to ethical considerations. However, it is worth noting that the brain circuitry involved in responding to primary and secondary reinforcers is not identical [17, 18], and different individuals within a species may have different associations with secondary reinforcers [11]. Ultimately, the ability to increase face validity by directly matching reinforcers across humans—who voluntarily consent to participate for research—and animals—whose entire existence falls within the research context—is always going to be a challenge.

### Predictive Validity

Predictive validity is also important, because if performance on a common currency task is not sensitive to the same interventions across species, it cannot be used for drug discovery. However, a focus on predictive validity has frequently resulted in the development of tasks which are only sensitive to ‘me-too’ pharmacological compounds (a term which refers to compounds that are chemically similar to an original, prototypical compound and therefore have the same mechanism of action as drugs already known to change behaviour in that task) [19–21]. Even tasks with high face validity, such as approach-avoidance conflict tasks used to investigate anxiety, may show predictive validity for one class of drug more consistently than another. For example, punishment-induced conflict tasks are sensitive to benzodiazepine administration, but SSRI administration does not consistently change responding, even though both classes of drug are effective in treating anxiety symptoms in humans [22, 23]. Ultimately, predictive validity depends on *what* is being predicted, which may be difficult to standardise because many of the things that matter to human patients (e.g. reduced ‘feelings’ of anxiety or more enjoyment of everyday activities) are not measurable in animals.

### Construct Validity

Finally, construct validity may be the most important contributor to translational validity, as tasks that measure the same construct should rely on common, evolutionarily preserved, cognitive, neural and biological mechanisms, thus ensuring that pharmacological agents should have similar effects on these tasks even when performed by different species [12]. However, it is difficult to prove that a given task demonstrates construct validity, as we have no access to the ground truth of which psychological phenomenon causes patterns of responding on any given task. Furthermore, by default, many tasks recruit several different psychological capabilities (e.g. working memory and reward learning in many reversal learning paradigms [24]), resulting in difficulty disentangling the respective contributions of each construct to performance.

## The Benefits of Common Currency Tasks

Despite the challenges, the key promise of common currency tasks is that by allowing the same endpoint to be measured in both preclinical and clinical drug development trials, the drug discovery pipeline in psychiatry will become more efficient [3, 19, 25, 26]. The failure of drugs in clinical trials for anxiety and depression can be partially attributed to lack of common endpoints. For instance, the promising pre-clinical trials of neurokinin-1 used forced-swim, tail suspension and stress paradigms [27, 28], whereas the human clinical trials, which failed, used symptom questionnaires [27].

Additionally, it is useful to have directly corresponding measures, rather than just two separate tasks for separate species that are purported to measure the same underlying construct. The primary benefit of this is that the data from different species can then be easily and directly compared, allowing any discrepancies on how species are performing the task to be detected (e.g. different patterns of accuracy in different conditions) and resolved. This direct comparison of results also allows changes in performance due to interventions or manipulations to be compared across species. For example, using a common currency task, Ironside et al. were able to show corresponding side-by-side plots of the probability of approach vs. avoidance responses for humans and non-human primates, allowing direct visual comparison of the behaviour of two different species on this task [29].

Another key advantage of valid common currency tasks is that they can allow us to obtain causal evidence for mechanisms, which is not possible using cross-sectional correlational designs: it is possible to directly manipulate genes, brain areas and protein expression in animals. For example, if we suspect that a particular gene is involved in fear extinction, we can only measure a correlation between genotype and behaviour on a task in humans. However, we can directly manipulate the expression and presence of that gene in animals, and then assess how this affects task performance, allowing stronger inference. Similarly, if we suspect that particular neural circuitry is involved in a behaviour (and, say, observe consistent neuroimaging patterns across species), we can use techniques such as inactivation and optogenetics to directly assess the effects of this circuitry on a task in animals. Crucially, the equivalent human work can generally only indirectly assess neural circuitry involvement via the blood-oxygen-level-dependent response in functional imaging, except in the rare cases where patients have lesions or implanted electrodes. Using common currency tasks in these experiments therefore allows us to draw a more direct causal conclusion about the mechanisms behind these symptoms, resulting in a better understanding of the pathophysiology. Integration of causal clinical, genetic and environmental information can ultimately build a more complete picture of the underlying mechanistic changes, and hence inform the most appropriate treatment strategies.

Furthermore, translational work in psychiatry is often stymied by the fact that many of the features we study—anhedonia, worry, intrusive thoughts—do not lend themselves to easy translation into animal work. Creating a veridical animal model of depression or psychosis is much more difficult than creating an animal model of, for example, cancer or diabetes, resulting in poor construct and face validity [20, 30]. Moreover, psychiatric disorders are highly heterogeneous and comorbid, with myriad possible presentations and symptoms, and as such are unlikely to be captured by a single animal model [31]. However, common currency tasks offer a partial alternative: we can study the *processes that may underlie* some of these complex symptoms, such as reward learning [10, 32], memory biases [33] or habit formation [34], instead, and use cross-species evidence to investigate our hypotheses. This type of process-based, or individual symptom-based approach, encompasses the search for ‘biomarkers’, ‘endophenotypes’ [35] or ‘research domains’ [36], which form key parts of the ‘experimental medicine’ approach to drug discovery [3].

Such process-based translational work might also allow drug discovery to be focused on symptoms that are not commonly targeted by treatments, but which are nevertheless of importance to patients. Many pharmacological agents target the ‘primary’ symptoms reported by patients—low mood in depression, delusions and hallucinations in schizophrenia—whilst other symptoms like concentration or memory receive less attention. For example, in both depression and schizophrenia, residual cognitive deficits are often still present after successful treatment of the primary symptoms with pharmacological agents [37–41]. Indeed, prior to a push to develop translationally valid tasks for cognition in schizophrenia, there was previously no mechanism for the FDA in the USA to approve a treatment for cognitive deficits in schizophrenia if said treatment did not *also* treat psychosis [26, 42, 43]. The development of further common currency tasks might enable a similar focus on overlooked symptoms in mood and anxiety disorders.

Finally, an indirect advantage of common currency tasks is that the need to ensure accessibility across species can enforce simplicity. As a result, these tasks tend to be more focused on a single underlying construct, which may facilitate a more precise measurement of specific processes without confounds. For example, a human neuropsychological task, the Wisconsin Card Sorting Test, is designed to measure set-shifting, but also implicitly requires the ability to perform successful visual matching. In this task, participants must sort cards into piles which share features such as shape, colour or number of items on the card, by learning over time which of these features should be matched for positive feedback. The target feature may change throughout the task, and successful performance following a change requires a ‘set-shift’: the participant must shift their attention and choices to another feature of the cards. Even once the target feature has been learnt, successful performance on this task requires visual matching—a participant must be able to identify the features present on the card in front of them and match these features to the features present on the four ‘piles’ at the top of the screen [5]. Thus, failure on the task could be due to impaired set-shifting (as is often inferred) or to impaired matching to sample. However, one common currency equivalent of this task—the intra-extra dimensional set shift task—was necessarily made simpler for use across species, but as a result, it is a more precise measure of set-shifting ability, which is less confounded by visual search ability as no visual matching is required [5].

## Three Examples of Common Currency Tasks

### Affective Bias

Recent work from our group has focused on translating an animal task that measures negative affective bias (a common feature of mood and anxiety disorders [20, 44]) into humans [10, 45]. This task, sometimes known as the ‘ambiguous-cue interpretation task’, was originally reported in 2004 [8]: rodents were trained to press a lever when they heard a tone that was associated with a positive event and to avoid pressing the lever when a tone was delivered that was associated with a negative event (70 dB white noise). Their affective bias was measured by how they subsequently responded to intermediate, non-reinforced tones: pressing the lever to intermediate tones on a lower proportion of trials indicated negative affective bias. Rodents experiencing a stressor (‘unpredictable housing’)—intended to create a state analogous to depression in humans—displayed increased negative affective bias. The possibility of measuring negative affective bias in rodents was an important advance, given that previous human work in this area had no corresponding animal paradigms.

**Fig. 2:**
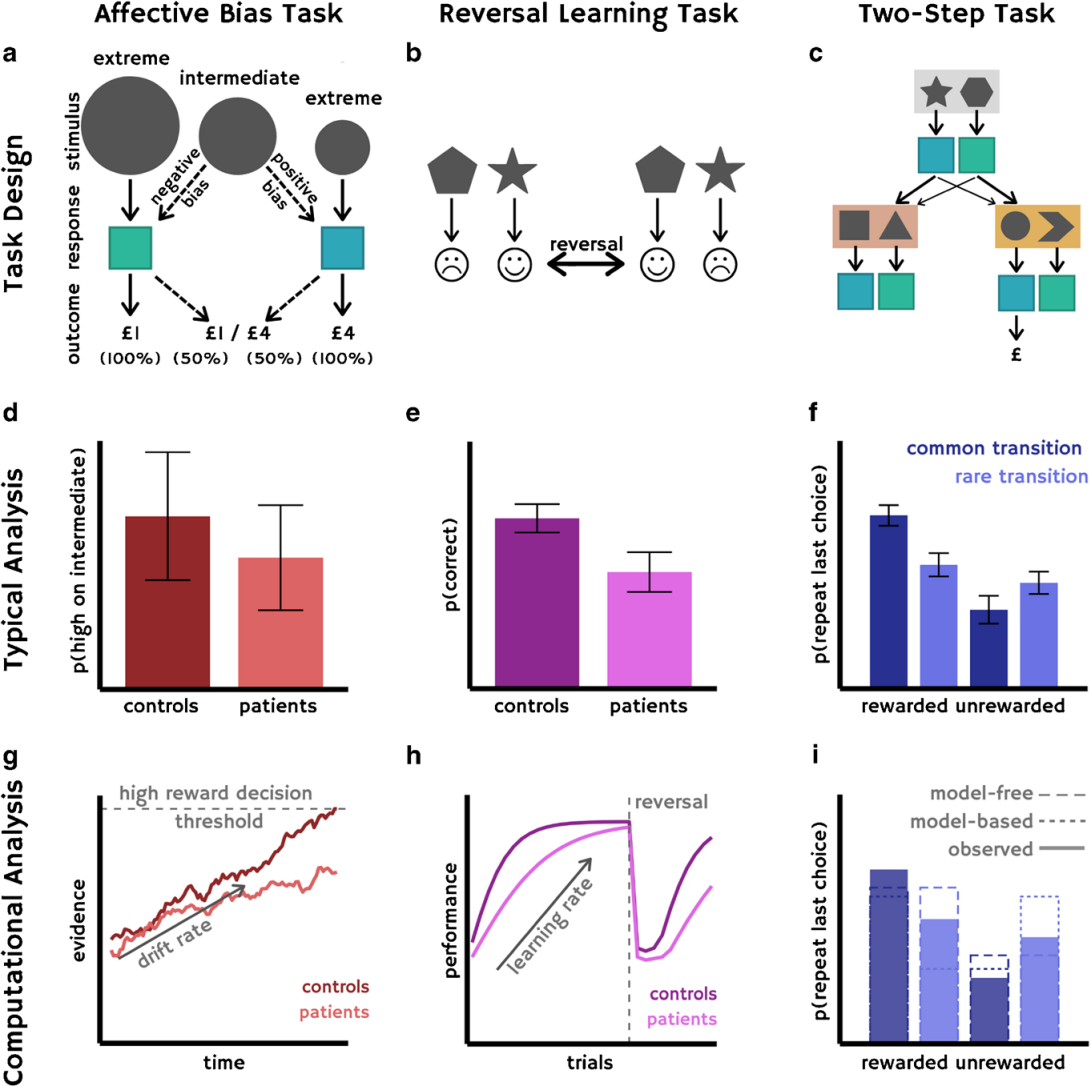
**a**–**c** The designs of the three types of cognitive task mentioned: the affective bias task, reversal learning task and two-step task. **d**–**f** Examples of how data is typically collapsed and analysed for these tasks. **g–i** Examples of the additional information that can be gained by taking a computational approach. **a** In the affective bias task, also known as the ‘ambiguous-cue interpretation task’, participants are first trained to press either the left or right button in response to the extreme stimuli (large or small circles in this example) which are 100% associated with either a £1 or £4 reward (associations counterbalanced across participants). In the test phase, during different trials, participants are shown either one of the original extreme stimuli or a novel, intermediate stimulus, to which they must respond by pressing the button associated with the stimulus they think it is closer to. On intermediate trials, there is a 50% chance of receiving a £1 or £4 reward. **d** Affective bias is operationalised here as the proportion of times participants press the button associated with the higher reward stimulus on intermediate stimulus trials. **g** An example of the drift rate, which can be estimated using a drift diffusion model (DDM), allowing us to account for participant accuracy and reaction times. In our work using this task [10], we found that patients with mood and anxiety disorders demonstrate a lower drift rate towards classifying the mid-tone as high reward. **b** In reversal learning tasks, participants typically choose between two stimuli on screen by pressing the corresponding button. One stimulus is associated with reward, indicated by a smiley face, and the other with punishment, indicated by a sad face. The contingencies are then reversed, so that the previously rewarded stimulus is now punished and vice versa. **e** The probability of participants choosing a correct (rewarded) choice. **h** The estimated learning rate; the shallower learning curve and greater latency before performance returns to high accuracy after a reversal is indicative of a slower learning rate in patients here. **c** In this example of a two-step task, participants start in one state (shown here in grey), and choose between two stimuli (star or hexagon), each of which result in a probabilistic transition (here, high probabilities are represented with a thicker arrow, and low probabilities—‘rare transitions’—with a thinner arrow) to a second-level state (either pink or orange), at which point they can choose between the two stimuli which are available to them in that state. Here, imagine that a participant chooses the star, and probabilistically moves to the orange state (on the right). They then choose the circle, which results in a reward. To obtain this reward again, the participant could perform in a ‘model-free’ way, without understanding the transitional structure of the stages, and simply choose the star again. However, this ‘model-free’ way of behaving is most likely to take them to the pink state, rather than the orange one. A ‘model-based’ choice would entail choosing the hexagon in state one, which is more likely to result in a transition to the desired orange state. When these choices are repeated over many trials, logistic regression or computational modelling can be used to demonstrate the extent to which participants behave in a ‘model-based’ way to seek out the best second state, rather than simply repeating actions which previously led to reward. **f** The probability of repeating the last trial, split by the outcome and transition type of the previous trial. **i** A computational modelling analysis of participant data (solid lines) can be used to estimate a ‘weight’ for each participant, which represents the extent to which they rely on model-based (dotted lines) and model-free (dashed lines) strategies

There have been a number of modifications to this task to remove confounds and improve validity [9, 20, 45–47]. Our direct human translation of this task used two differently sized rewards (Fig. 2) and also analysed the data using the same computational modelling approach adopted for the animal task [45]. This task can also be performed with visual rather than auditory stimuli [48] in humans, which may be more ethologically relevant even though it shows lower face validity [49].

There are a number of strengths of this common currency task: firstly, the task can be performed by diverse species including starlings [50], honeybees [51], drosophila [52] and macaques [53], and secondly, there is an associated computational model that captures performance on this task [45]. The computational model used is a version of a drift diffusion model [54, 55]. Using this model identifies (putatively mechanistic) latent parameters, rather than just summary statistics such as ‘mean accuracy’, which can be compared between species and across manipulations. However, whilst this task seems to have good construct validity, it has not shown strong predictive validity in acute-administration antidepressant studies in animals thus far, though this may relate to the delayed-onset mechanism of action of most commonly prescribed antidepressants [20].

### Reversal Learning

Another recent paper that used a common currency task to collect complementary data across-species focused on the CACNA1C gene (which encodes a subunit of a type of voltage-gated calcium channel) and reversal learning [56]. Variants of this gene have been linked to risk for schizophrenia and bipolar disorder [57, 58]. The authors demonstrated that reversal learning (Fig. 2) was impaired in humans with two different risk alleles in the CACNA1C gene, and also in rats who were heterozygote knockouts for CACNA1C. They subsequently demonstrated in data from post-mortem tissue that the human risk alleles result in lower expression of BDNF (brain-derived neurotrophic factor) in the prefrontal cortex. Similarly, the rat heterozygote knockouts also displayed reduced prefrontal BDNF expression, according to results from both in situ hybridization and qPCR. The authors subsequently recommend that reversal learning tasks should be used in translational research targeting voltage-gated calcium channels.

Using a common currency task in this study allowed the authors to demonstrate convincingly in both humans and rodents that variants in the CACNA1C gene result in impairments in reversal learning, and they were able to use complementary methods in animals and humans to demonstrate that this may be underpinned by reduced BDNF in the prefrontal cortex. These converging sets of evidence are more convincing than data from either species would be alone. Furthermore, if this study had just been performed in humans, it would not be possible to directly manipulate the gene of interest, and instead, the conclusions would have to rely on associations between genotype and task performance.

However, the reversal-learning task used differed substantially between animals and humans. In particular, in the human task, the stimuli were coloured squares, and in the animal task, these were different shapes. In the human task, rewards were ‘smiley’ faces and 1p monetary gain, and punishments ‘frowny’ faces and 1p monetary loss, which were passively received. By contrast, in the animal task, the reward was 10% sucrose solution which was actively obtained from the magazine, and the punishment was a 10-s ‘time-out’ periods. In the human task, reversals occurred after 7–11 trials, whereas in the animal task, there was only one reversal, which occurred after 2 days of > 80% accuracy on the task. The level of training also differed—animals were trained on both the procedure for reward collection (collecting sucrose from the magazine) and the association of a nose-poke action with a reward, whereas humans were not trained. The variable from the human task that was compared between genotype was accuracy after the first reversal along with total earnings, whereas the variable from the rodent task that was used was percentage of animals of each genotype that completed each experimental condition. Whilst these differences may be more related to face validity than construct validity, future work may focus on aligning these paradigms more closely.

### Goal-Based Decision-Making

Another study that combines some of the strengths from the first and second studies is a back-translation of the two-step task (Fig. 2), commonly used to demonstrate disrupted goal-based decision making in OCD [59, 60], for use in rodents [61]. Computational models akin to those used in humans were fitted to rodent behaviour, and it was demonstrated that muscimol inactivation of either the dorsal hippocampus or the OFC caused a reduction in the use of model-based reinforcement learning. This allowed the authors to conclude that these brain regions causally contribute to this type of learning, whereas MRI could only demonstrate an association. The use of modelling in conjunction with a common currency task also allowed the researchers to compare behaviour between species in a more technical way: they stated in their discussion that the lack of observed model-free planning in rats compared with humans may be due to the increased training that rats received on the task. It is possible that the number of hours of training rodents receive could be adjusted until computational analyses, performed on choices generated by both humans and rodents, show no difference in the extent to which these species are using model-free learning strategies. This may ensure more optimal construct validity.

## Future Directions and Recommendations

The advent of novel technological solutions has brought new options to the development of common currency tasks. Technologies such as the use of a touch screen [49, 62] allow all species to have the same access to instructions and training, whilst offering standardisation and higher throughput. Crucially, both stimuli and responses can be in the same modality between species, and touch-screens may ensure that all animals are using the same strategy to complete the task [49]. The use of virtual reality [7] allows for human participants to be placed in environments closer to those used in classical animal tasks—such as mazes—without the corresponding needs for space and sophisticated ethical controls. Virtual reality versions of the Morris water maze have been used in both stress and schizophrenia research in humans [63, 64]. Recent work in humans has also used virtual reality to create ethical, and precisely-controlled, threatening and non-threatening contexts [65]. Furthermore, the use of virtual reality has also been found to be beneficial in animals, as well as humans, either to precisely control the environment or to reduce animal motion when using techniques such as two-photon imaging or fMRI [66–68].

It has also been acknowledged that using computational modelling could allow more of a mechanistic understanding of behaviour on tasks [69]. Typically, behavioural tasks are analysed using summary statistics (Fig. 2d-f), which may capture differences between groups or relevant correlations, but are atheoretical. Generative computational models contain not only tractable summaries of data, but also contain within them hypotheses about the (hidden) processes which led to the generation of the observed behaviour (such as, for example, learning rate, which cannot be directly observed—only inferred from a set of responses collected over time). Methods for adjudicating between different models enable researchers to directly assess the evidence for different hypothesised data-generating processes, and differences in computational parameters may reflect changes to the mechanisms which are hypothesised to generate behaviour, such as learning rate, behavioural noise or prior beliefs about the world. Notably, two of the papers discussed above in the example section used a common currency task with an associated computational model (Fig. 2g-i) [10, 61]. The use of computational modelling allows hypotheses about the strategies used by different species or participants to be quantitatively tested, and the fit of different models (representing different strategies) compared. It is yet to be seen whether using computational modelling improves translational efficiency in psychiatric research, but it may be a valuable avenue for exploration.

In Table 1, we therefore provide a summary of potential aspects that may influence validity that researchers should consider when designing or translating tasks. We recommend that future common currency tasks, rather than trying to make tasks precisely identical in all the ways shown, ascertain which elements of the task should match in order to provide the highest likelihood that the underlying cognitive strategies and neural mechanisms are the same [26, 49]. Not only may species be using different strategies to perform the same apparent behaviour [3, 12, 70], animals may also be performing a different behaviour altogether (resulting in poor construct validity, despite accompanying good face validity).

**Table 1:** Possible aspects of the tasks that are ‘common’ in common currency tasks—for consideration when designing new tasks

Common aspects	Type of validity	Notes
Demographics of sample	Face/construct	Many animal studies only use males [71]—is this appropriate for the research question? Are humans only recruited if they fit into certain demographics (age, medication)?
Developmental stage	Face/construct	Is the same developmental stage used in both human and animal research? e.g. adolescent vs adult
Task difficulty	Construct	Does the task need to be simpler for animals to achieve the same level of performance? Do overtrained animals perform better than humans?
Task duration	Face/construct	Do different species need different task durations? i.e. do humans get bored faster/produce more varied behaviour so more data points are needed for accurate inference? Do animals need many short sessions of a task, whereas humans can perform the task in one longer session?
Motivation	Construct	Are animals water restricted to maximise their desire for (water) reward? Are humans reimbursed more for good performance?
Training		
Instructed	Face/construct	Are the instructions verbal?
Overtraining	Face/construct	Animals are frequently overtrained on tasks, whereas humans are usually not
Stimulus presentation		
Modality	Face/construct	Visual, aural? Would construct validity be achieved better if stimuli are different between species?
Actual stimuli	Face/construct	E.g. tones may be adjusted for different species’ hearing ranges
Response	Face/construct	E.g. do animals and humans both press buttons, or do animals enter a nose-poke?
Feedback		
Classification	Face/construct	Often, animals receive primary reinforcers such as sucrose or electrical shocks, and humans receive points or money
Actual feedback	Face/construct	Even if both primary reinforcers, feedback may still differ: e.g. white noise in humans, and electric shocks in animals
Strategy	Construct	Are animals and humans using the same ‘strategy’ to complete the task? For example, animals and humans may rely to different extents on spatial strategies in the Morris water maze and the virtual-reality human equivalent [16]
Data preprocessing	Construct	Is data quality assessed in the same way between species? Are data cleaned in the same way?
Analysis	Construct	Are the primary outcome measures the same? Are they calculated in the same way?
Behavioural performance	Construct	Is behavioural performance (e.g. patterns of accuracy) similar between species?
Neural basis	Construct	Are homologous brain areas and circuits implicated in the performance of this task between species?
Sensitivity to symptoms	Face/construct/predictive	Is behaviour on the task sensitive to psychiatric symptoms, e.g. do animal models of anhedonia demonstrate a measurable change from healthy animals in the same way anhedonic humans perform differently to healthy controls?
Effects of interventions	Predictive	Do pharmacological agents have the same effects on both human and animal behaviour/neural activity in the task?

In addition to the task design, it is also important to consider the experimental subjects. Most research in animals is performed using males [71] and is also often restricted to specific inbred strains, which are not necessarily representative of wild-type animals [72]. Limiting translational research to highly standardised and constrained populations is likely to reduce the generalisability of findings to humans. For instance, in human psychiatry research, there are significant gender differences between the prevalence of different disorders, with mood and anxiety disorders being more prevalent in women than in men [73]. Limiting animal work to male subjects may drive differences between preclinical and clinical findings and increase the chance of translational failure. Similarly, many psychiatric disorders are thought to be polygenic and have epigenetic influences, factors which are hard to account for and study using a genetically close-to-identical sample [74].

We also recommend that evidence indicating the extent of construct validity is summarised in papers presenting common currency tasks, whether this is behavioural, neural or otherwise. Whilst face validity may be easy to assess by comparing the methods used across species, assessing construct validity is harder—and face validity does not necessarily entail construct validity, as described above.

A ‘multifactorial’ approach is also recommended, including both behavioural and neural measures, as this could increase confidence in translational results [3, 11, 19]. In particular, as fMRI becomes a more common concomitant of human research, fMRI in animals shows increasing promise as a directly translatable measure of the neural effects of new pharmacological agents on common currency tasks [19].

Finally, we recommend that future research and development of common currency tasks should be bi-directional: basic research should be used to inform clinical practice, and clinical observations can inform basic research. Both translation and back-translation should be iterated over in order to obtain tasks that are truly translationally valid. This work will ensure that the promise of common currency tasks is truly achievable.

## Conclusions

In this review, we have discussed the definitions of common currency tasks and the aspects of tasks which may be consistent across animals and humans. We have also highlighted several benefits of common currency tasks: the most important of which is that they may alleviate the ‘bottleneck’ in drug development work. Three recent examples using common currency tasks are discussed in detail, with their strengths and limitations. We conclude by offering several recommendations for future work: including focus on construct rather than face validity, use of multifactorial methods and novel technological approaches, and the use of computational models. If progress in this field is sustained, common currency tasks may offer a window of opportunity for significant advances in translational work, hopefully heralding a new period of psychiatric drug discovery.
